# Direct-Current Electric Field Distribution in the Brain for Tumor Treating Field Applications: A Simulation Study

**DOI:** 10.1155/2018/3829768

**Published:** 2018-02-22

**Authors:** Yung-Shin Sun

**Affiliations:** Department of Physics, Fu-Jen Catholic University, New Taipei City 24205, Taiwan

## Abstract

Tumor Treating Fields (TTFields) in combination with chemotherapy and/or radiotherapy have been clinically reported to provide prolonged overall survival in glioblastoma patients. Alternating electric fields with frequencies of 100~300 kHz and magnitudes of 1~3 V/cm are shown to suppress the growth of cancer cells via interactions with polar molecules within dividing cells. Since it is difficult to directly measure the electric fields inside the brain, simulation models of the human head provide a useful tool for predicting the electric field distribution. In the present study, a three-dimensional finite element head model consisting of the scalp, the skull, the dura, the cerebrospinal fluid, and the brain was built to study the electric field distribution under various applied potentials and electrode configurations. For simplicity, a direct-current electric field was used in the simulation. The total power dissipation and temperature elevation due to Joule heating in different head tissues were also evaluated. Based on the results, some guidelines are obtained in designing the electrode configuration for personalized glioblastoma electrotherapy.

## 1. Introduction

Glioblastoma, or glioblastoma multiforme (GBM), is the most aggressive malignant brain tumor, having an incidence of about 4.43 out of 100,000 persons in the United States [1]. Initial symptoms of glioblastoma include headaches, personality changes, epilepsy, nausea, and hemiparalysis, and unconsciousness may be the sign of exacerbation [2]. Surgery, if applicable, is the first step of GBM treatment, and then radiotherapy and chemotherapy could follow. For radiotherapy, patients who received total radiation doses of 50~60 Gy were reported to have 1.6~2.3 times longer life expectancy compared with those receiving no radiotherapy [3]. For chemotherapy, patients given standard radiation plus temozolomide (an oral chemotherapy drug) survived a median of 14.6 months compared to 12.1 months for those receiving radiation alone [4]. Recently, immunotherapy and gene therapy have also been applied in GBM treatment [5–7]. Although various novel therapies were clinically reported to extend patient survival rate, glioblastoma is considered incurable, with a medium survival period of 14.6 months and a two-year survival rate of 30% [8].

As a new therapeutic technology for treating GBM, Tumor Treating Fields (TTFields) suppress the growth of cancer cells by applying alternating electric fields (EFs) with frequencies of 100~300 kHz and magnitudes of 1~3 V/cm. EFs were shown to play important roles in various physiological processes such as cell division and wound healing [9, 10]. Direct-current (dc) or alternating current (ac) EFs were reported to induce collective and directional migration of adherent cells, phenomena termed electrotaxis [11–13]. Compared to radiotherapy or chemotherapy, TTFields are considered safer and produce fewer side effects. Kirson et al. found that TTFields inhibit cancerous cell growth via an antimicrotubule mechanism of action [14]. In brief, applied alternating EFs interact with polar molecules (i.e., microtubules) within dividing cells, leading to the disruption of microtubule spindle formation during the mitotic phase [15]. Such TTFields have also been shown to arrest* in vitro* cell proliferation of various cancers in frequency- and dose-dependent manners. For example, the optimal frequency was 100 kHz for mouse melanoma, 150 kHz for human breast carcinoma, and 200 kHz for rat glioma [14]. And to kill 80% of cancer cells, the required intensity was 1.3 V/cm for mouse melanoma, 2.2 V/cm for rat glioma, 2.7 V/cm for human non-small cell lung carcinoma, and 3 V/cm for human breast carcinoma [14]. TTFields were considered most effective when applied for 24 hours to cells which undergo mitosis and are oriented roughly along the direction of EFs [16].

Clinically, when combined with chemotherapy, TTFields worked well in treating various cancers. For example, Gharaee et al. demonstrated that TTFields of 150 kHz coadministrated with doxorubicin can be used as an alternative strategy for breast cancer therapy to improve the effects of the drugs and increase the sensitivity of cancer cells [17]. Giladi et al. investigated the response of Lewis lung carcinoma and KLN205 squamous cell carcinoma in mice treated with TTFields in combination with pemetrexed, cisplatin, or paclitaxel and concluded that combining TTFields with these therapeutic agents enhanced treatment efficacy in comparison with the respective single agents and control groups in all animal models [18]. Moreover, TTFields in combination with paclitaxel and gemcitabine were reported therapeutically effective against ovarian and pancreatic cancers, respectively [19, 20]. The Optune™ (formerly NovoTTF™-100A) system developed by Novocure (https://www.novocure.com) is a portable medical device used to deliver low-intensity (>0.7 V/cm), intermediate-frequency (200 kHz) alternating EFs to the brain via noninvasive transducer arrays attached to the shaved scalp of glioblastoma patients. It has been approved for the treatment of GBM in the European Union, Switzerland, Australia, Israel, Japan, and the United States. As a pilot clinical trial, 10 patients with recurrent glioblastoma were treated with TTFields as a monotherapy. No device-induced serious side effects were observed after more than 70 months of cumulative treatment except mild to moderate contact dermatitis due to electrodes [14]. In 2012, a phase III trial of NovoTTF (20–24 h/day) in the treatment of patients with recurrent glioblastoma was conducted. Although no improvement in overall survival was observed, efficacy and activity of this chemotherapy-free treatment system appeared comparable to traditional chemotherapy [21]. Wong et al. treated a series of patients with NovoTTF-100A and bevacizumab alone or in combination with a regimen consisting of 6-thioguanine, lomustine, capecitabine, and celecoxib (TCCC) and found that, compared to the former group, the latter group exhibited a trend for prolonged overall survival [22]. Recently, an international, multicenter, prospective, and randomized phase III trial (EF-14) was conducted for testing the efficacy of combining TTFields with standard chemotherapy of temozolomide (TMZ) in 695 newly diagnosed GBM patients. The results demonstrated better progression free survival and overall survival in patients treated with a combined therapy of TTFields and TMZ compared with those receiving TMZ alone [23].

In a TTField-based device, transducer arrays of electrodes are placed on the patient's shaved scalp. As mentioned earlier, it is of importance to be able to deliver alternating EFs of desired intensity to the tumor inside the brain. However, with exosomatic applied TTFields, it is difficult to measure the distribution of the EFs inside the brain. The only one intracranial measurement conducted by Kirson et al. indicated that effective (1~2 V/cm) TTFields could be generated at the center of the brain by applying 50 V to surface electrodes placed on the scalp [14]. Therefore, simulation models of the human head provide a useful tool for predicting the EF distribution inside the brain. It may also help to personalize the treatment by adjusting the positions of electrodes to better treat tumors at certain locations with desired intensities. In this study, I built a three-dimensional (3D) head model consisting of the scalp, the skull, the dura, the cerebrospinal fluid, and the brain. Each type of tissue has its own conductivity, relative permittivity, density, and heat capacity. Using the finite element method (FEM) and the commercial software COMSOL Multiphysics, I simulated the distribution of EFs inside the brain under different electrode configurations and applied intensities. For simplicity, a direct-current electric field was used in the simulation. The total power dissipation due to Joule heating in different head tissues was also evaluated. The results are believed to be helpful in designing the electrode configuration for personalized GBM electrotherapy.

## 2. Materials and Methods

To investigate how the magnitudes of applied voltage affect the intensities of EFs generated inside the brain, a dc module instead of ac module is used. Within various head tissues, the EF resulting from a constant dc can be treated as quasi-stationary over time. By flowing a constant dc through volume conductors of homogeneous and isotropic electrical properties, steady dcEFs are generated. The distribution of electric potential (*V*) is then governed by the Laplace equation, ∇^2^*V* = 0, with appropriate boundary conditions. In the Dirichlet boundary condition, a fixed scalar potential (i.e., the applied voltage) is specified on the surface of the model.

### 2.1. The Head Model

**Figure 1:**
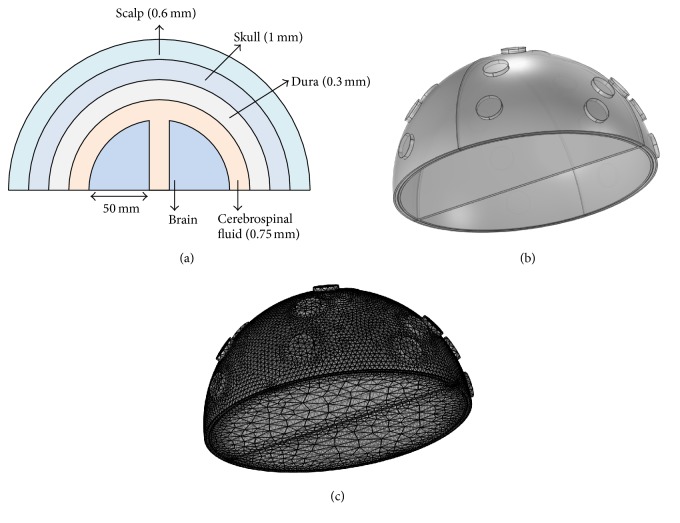
(a) The geometry of the head (not to scale). (b) The head model constructed in COMSOL. (c) The finite element mesh constructed in COMSOL.

A 3D finite element head model was built using the software COMSOL Multiphysics (Version 4.4, MI, USA). The geometry of the head is shown in Figure 1(a) [24]. The scalp, consisting of five layers, has a thickness of 0.6 mm. The skull, supporting the structures of the face and providing a protective cavity for the brain, has a thickness of 1 mm. The dura, having a thickness of 0.3 mm, is a thick membrane surrounding the brain and spinal cord. The cerebrospinal fluid, a clear, colorless body fluid acting as a cushion for the brain, has a thickness of 0.75 mm. The brain, having a radius of 50 mm, is composed of 40% of grey matter and 60% of white matter. As shown in Figure 1(b), the whole head is modeled as a half sphere with a radius of 52.65 mm.  Figure 1(c) shows the finite element mesh made of 220,620 tetrahedral elements, 67,626 triangular elements, 2,642 edge elements, and 180 vertex elements.

### 2.2. Tissue Properties

The electrical properties of different head tissues are listed in Table 1. For simplicity, all tissues were modeled as homogenous, isotropic conductors with constant conductivities and relative permittivities throughout. The conductivities of the scalp, the skull, the dura, the cerebrospinal fluid, and the brain are 0.00105, 0.0529, 0.502, 2, and 0.108 Sm^−1^, respectively. The relative permittivities of these five tissues were 1100, 295.5, 290, 109, and 1578, respectively. To evaluate temperature elevation due to Joule heating, the densities and heat capacities of different head tissues are also listed in Table 1. The densities of the scalp, the skull, the dura, the cerebrospinal fluid, and the brain are 1109, 1543, 1174, 1007, and 1043 kgm^−3^, respectively. The heat capacities of these five tissues were 3391, 1793, 3364, 1096, and 3628, respectively.

### 2.3. Simulation Conditions

The head model is used to simulate the distribution of endogenous dcEF inside the brain. The Electric Currents (ec) module of the COMSOL Multiphysics is used to solve the steady-state EF distribution. The following equations are used:

(1) ∇·J=Q,J=σE+Je,E=−∇V.

**Figure 2:**
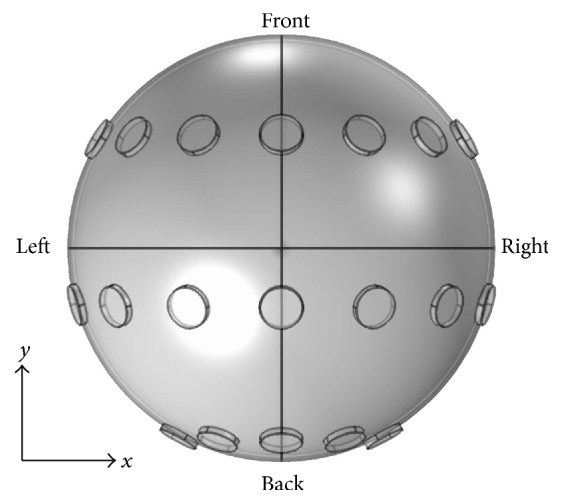
The configuration of the electrodes used in the head model.

In these equations, **J** is the current density, **Q** is the electric charge, *σ* is the electrical conductivity, **E** is the electric field, **J**_**e**_ is the externally generated current density, and *V* is the electric potential. The configuration of the electrodes is shown in Figure 2. As indicated, there are 19 copper electrodes in total, with 7 in the front, 7 in the middle, and 5 in the back. Each electrode is assigned a potential *V* (positive electrode, shown as “+”), a ground (negative electrode, shown as “−”), or a nude (neither positive nor negative). The* x-*,* y-*, and* z*-axes of the head model are also illustrated. The endogenous dcEF is shown in a cross-sectional view (of the* xy*,* yz*, or* zx* plane) and a line profile (along a given direction). The distributions of dcEFs inside the brain under different positive/negative electrode configurations and applied dcEF strengths are investigated. To achieve personalized treatment for GBM, various positive/negative electrode configurations are tested to focus the dcEFs on certain locations. Finally, the total power dissipation and temperature elevation due to Joule heating in different head tissues are evaluated using the densities and heat capacities of these tissues. Hyperpyrexia due to excessive Joule heating can cause serious side effects such as headache and burn.

## 3. Results and Discussion

**Figure 3:**
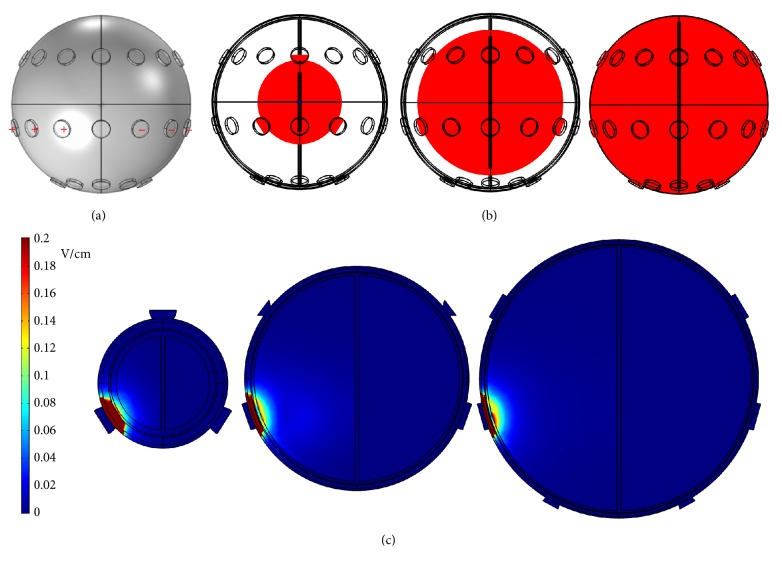
(a) The electrode configuration used to apply a potential of 0.5 V. (b) The* x*-*y* plane cross-sectional views at* z* = 47, 33, and 13 mm from left to right. (c) The* x*-*y* plane cross-sectional views of the endogenous dcEFs corresponding to (b).

**Figure 4:**
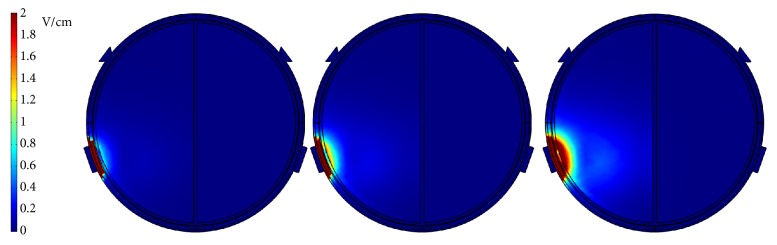
The* x*-*y* plane cross-sectional views (*z* = 33 mm) of the endogenous dcEFs with applied potentials of 2.5, 5, and 10 V from left to right.

First, a potential of 0.5 V was applied to the left 3 electrodes of the middle array, and the right 3 electrodes of the middle array were grounded, as shown in Figure 3(a). The* x*-*y* plane cross-sectional views of the endogenous dcEFs are shown in Figure 3(c) (*z* = 47, 33, and 13 mm from left to right in Figure 3(b)). In all 3 subfigures, the dcEF strengths are the highest near the electrodes, but these values decrease rapidly as they cross the dura and the cerebrospinal fluid. dcEFs of only around 0.1 V/cm are attained near the outmost layer of brain and they are partially localized in very narrow regions. This dcEF strength is obviously not enough for GBM treatment, and a higher applied voltage is required.  Figure 4 shows the* x*-*y* plane cross-sectional views (*z* = 33 mm) of the endogenous dcEFs with applied potentials of 2.5, 5, and 10 V (resp., from left to right). The electrode configuration is the same as that in Figure 3(a). Under an applied voltage of 2.5 V, dcEFs of 0.4~1 V/cm are generated in the cerebrospinal fluid and the outmost layer of the brain. Similar results are observed when the applied potential is increased to 5 V: dcEFs of 0.8~1.4 V/cm are generated in the cerebrospinal fluid and the outmost layer of the brain. At an applied potential of 10 V, dcEFs of >1.8 V/cm are attained in similar regions. In all 3 subfigures, the dcEFs are partially localized near the electrodes from this point of view (i.e., the* x*-*y* plane cross-sectional view). I will now check the distributions of dcEFs from different cross-sections.

**Figure 5:**
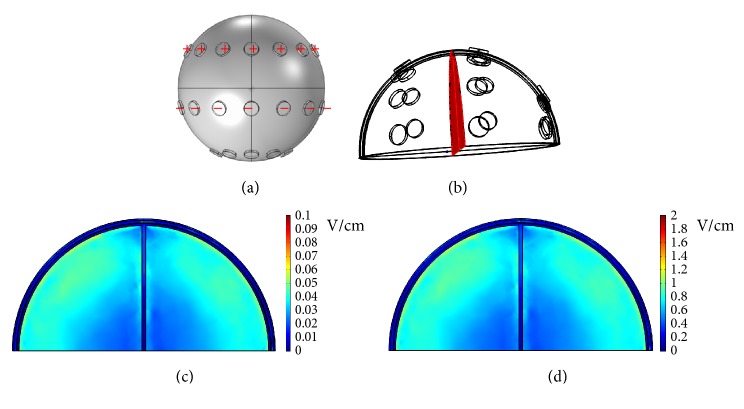
(a) The electrode configuration used to apply potentials of 5 V and 100 V. (b) The* x*-*z* plane cross-sectional view at* y* = 0 mm. (c) The* x*-*z* plane cross-sectional view of the endogenous dcEF at an applied potential of 5 V. (d) The* x*-*z* plane cross-sectional view of the endogenous dcEF at an applied potential of 100 V.

**Figure 6:**
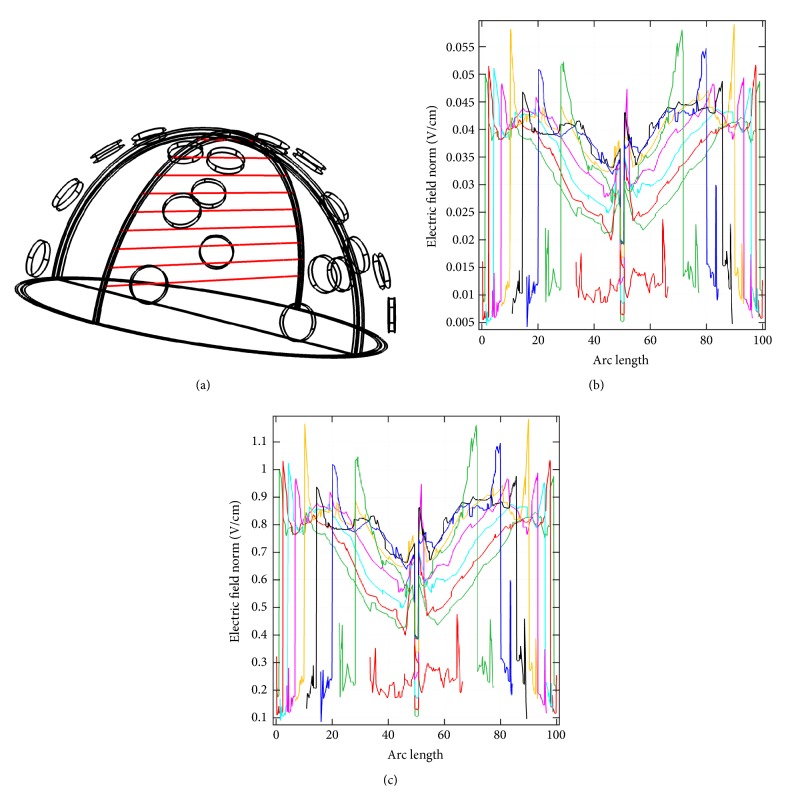
(a) Nine lines along the* x-*axis in the plane shown in Figure 5(b). (b) The dcEF profiles at an applied potential of 5 V along these lines. (c) The dcEF profiles at an applied potential of 100 V along these lines.

Figure 5(a) shows another electrode configuration where potentials were applied to all electrodes of the front array, and all electrodes of the middle array were grounded. At an applied voltage of 5 V, the* x*-*z* plane cross-sectional view (*y* = 0 mm, as shown in Figure 5(b)) of the endogenous dcEF is illustrated in Figure 5(c). The dcEFs are distributed more or less uniformly throughout the cross-section and have strengths of only 0.025~0.04 V/cm. These intensities are not enough for GBM treatment. When the applied potential is increased to 100 V, the dcEF distribution is shown in Figure 5(d). The dcEF strengths increase to 0.5~1 V/cm, suitable for electrotherapy applications. These values are close to those reported by Kirson et al.: an applied potential difference of 50 V could generate a TTField of 1~2 V/cm at the center of the brain [14]. Nine lines along the* x*-axis in that plane are shown in Figure 6(a). The dcEF profiles at applied potentials of 5 V and 100 V along these lines are illustrated in Figures 6(b) and 6(c), respectively. At 5 V, the dcEF strengths go from small (around 0.005~0.01 V/cm in the first 5 mm) to large (around 0.025~0.04 V/cm in the middle range) and then to small again (around 0.005~0.01 V/cm in the last 5 mm). Similarly at 100 V, the dcEF strengths go from small (around 0.1~0.3 V/cm in the first 5 mm) to large (around 0.5~1 V/cm in the middle range) and then to small again (around 0.1~0.3 V/cm in the last 5 mm).

**Figure 7:**
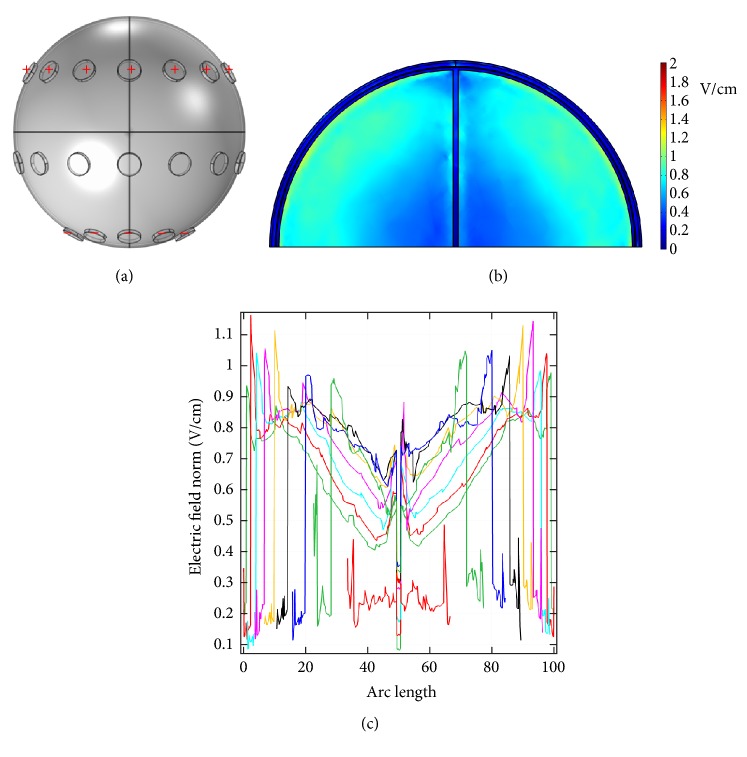
(a) The electrode configuration used to apply a potential of 100 V. (b) The* x*-*z* plane cross-sectional view (*y* = 0 mm) of the endogenous dcEF. (c) The dcEF profiles corresponding to 9 lines in the* x*-*z* plane cross-section.

**Figure 8:**
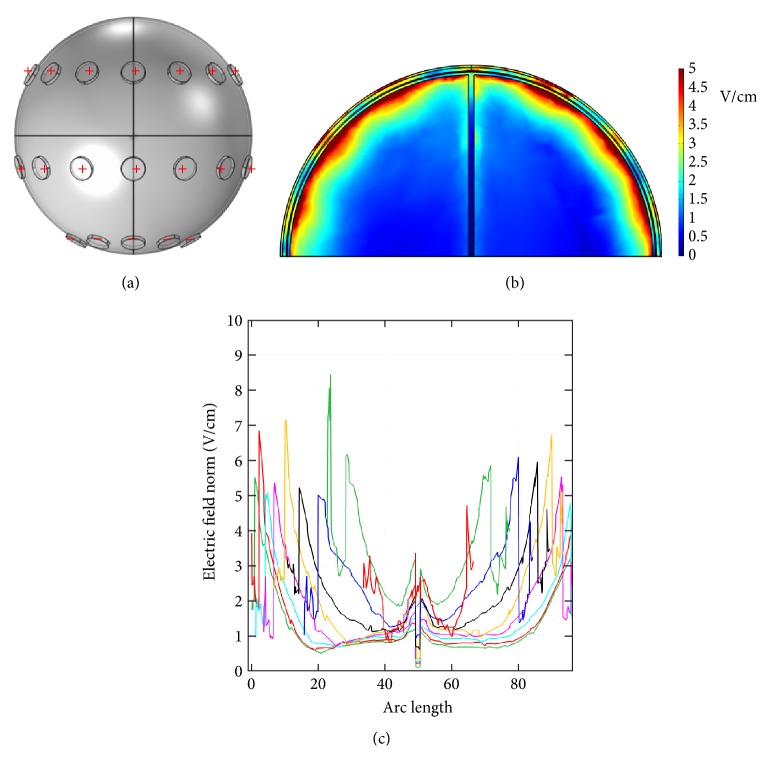
(a) The electrode configuration used to apply a potential of 100 V. (b) The* x*-*z* plane cross-sectional view (*y* = 0 mm) of the endogenous dcEF. (c) The dcEF profiles corresponding to 9 lines in the* x*-*z* plane cross-section.

Figure 7(a) shows another electrode configuration similar to that in Figure 5(a). Potentials were applied to all electrodes of the front array, and all electrodes of the back array were grounded. At an applied potential of 100 V, Figure 7(b) displays the dcEF distribution in the* x*-*z* plane cross-section (*y* = 0 mm), and Figure 7(c) shows 9 dcEF profiles along the* x*-axis in that plane. As indicated, the dcEFs are distributed more or less uniformly throughout the cross-section and have strengths of 0.5~1 V/cm, similar to those in Figures 5(d) and 6(c). The electrode configuration is then changed to that in Figure 8(a), where all electrodes of the front and middle arrays were assigned potentials and all others were grounded. At an applied voltage of 100 V, the dcEF distribution in the* x*-*z* plane cross-section (*y* = 0 mm) indicates that dcEFs are localized mainly along the boundary of the brain, as shown in Figure 8(b). Nine line profiles along the* x*-axis in that plane show that the dcEF strengths go from large (around 3~5 V/cm in the first 10 mm) to small (around 1~3 V/cm in the middle range) and then to large again (around 3~5 V/cm in the last 10 mm), as displayed in Figure 8(c).

**Figure 9:**
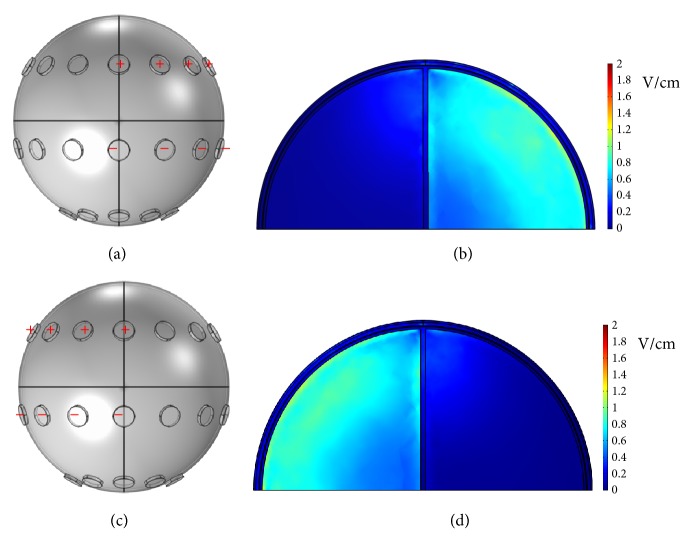
(a) The electrode configuration where the right 4 electrodes of the front array are assigned potentials and the right 4 electrodes of the middle array are grounded. (b) The* x*-*z* plane cross-sectional view (*y* = 0 mm) of the endogenous dcEF corresponding to (a). (c) The electrode configuration where the left 4 electrodes of the front array are assigned potentials and the left 4 electrodes of the middle array are grounded. (d) The* x*-*z* plane cross-sectional view (*y* = 0 mm) of the endogenous dcEF corresponding to (c).

**Figure 10:**
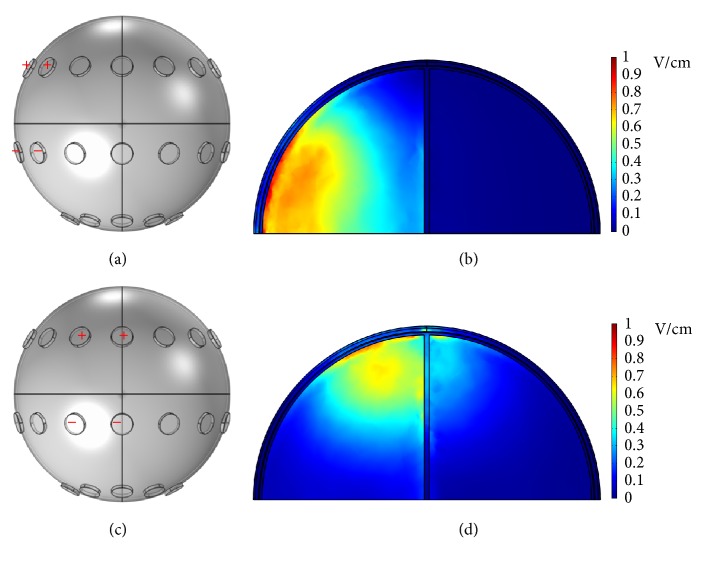
(a) The electrode configuration where the left 2 electrodes of the front array are assigned potentials and the left 2 electrodes of the middle array are grounded. (b) The* x*-*z* plane cross-sectional view (*y* = 0 mm) of the endogenous dcEF corresponding to (a). (c) The electrode configuration where the middle 2 electrodes of the front array are assigned potentials and the middle 2 electrodes of the middle array are grounded. (d) The* x*-*z* plane cross-sectional view (*y* = 0 mm) of the endogenous dcEF corresponding to (c).

**Figure 11:**
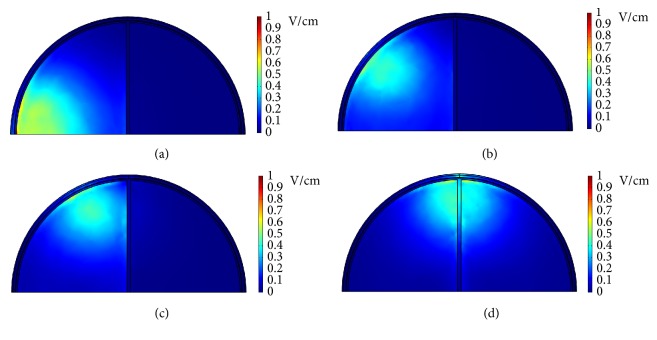
The* x*-*z* plane cross-sectional views (*y* = 0 mm) of the endogenous dcEFs corresponding to the electrode configurations where (a) the leftmost electrodes of the front and middle arrays are assigned potentials and grounded, respectively; (b) the second electrodes from the left of the front and middle arrays are assigned potentials and grounded, respectively; (c) the third electrodes from the left of the front and middle arrays are assigned potentials and grounded, respectively; (d) the center electrodes of the front and middle arrays are assigned potentials and grounded, respectively.

Next, different electrode configurations are tested to see whether it is possible to focus the dcEFs on certain locations.  Figure 9(a) shows the electrode configuration where the right 4 electrodes of the front array were assigned potentials and the right 4 electrodes of the middle array were grounded. At an applied potential of 100 V, the dcEF distribution in the* x*-*z* plane cross-section (*y* = 0 mm) is displayed in Figure 9(b). As illustrated, the dcEFs are distributed more or less uniformly throughout one-half of the cross-section where positive and negative electrodes are assigned. The dcEF strengths range from 0.5 to 1 V/cm. When the left 4 electrodes of the front array were assigned potentials and the left 4 electrodes of the middle array were grounded (as shown in Figure 9(c)), the* x*-*z* plane cross-sectional view indicates that the dcEFs are distributed more or less uniformly throughout the other half of the cross-section. In the electrode configuration shown in Figure 10(a) where the left 2 electrodes of the front array are assigned potentials and the left 2 electrodes of the middle array are grounded, the dcEF distribution in the* x*-*z* plane cross-section (*y* = 0 mm) is displayed in Figure 10(b). The dcEFs, having strengths of 0.5~0.8 V/cm, are localized in the leftmost one-quarter of the cross-section where positive and negative electrodes are assigned. When the electrodes are switched to the next 2 electrodes on the right (see Figure 10(c)), the* x*-*z* plane cross-sectional view indicates that the dcEFs are localized in the top half of the second one-quarter of the cross-section, as displayed in Figure 10(d).  Figure 11 shows the* x*-*z* plane cross-sectional views (*y* = 0 mm) of the endogenous dcEFs when (a) the leftmost electrodes of the front and middle arrays are assigned potentials and grounded, respectively; (b) the second electrodes from the left of the front and middle arrays are assigned potentials and grounded, respectively; (c) the third electrodes from the left of the front and middle arrays are assigned potentials and grounded, respectively; (d) the center electrodes of the front and middle arrays are assigned potentials and grounded, respectively. As clearly shown, when the positive and negative electrodes move from left to right, the dcEFs shift accordingly. These dcEFs have similar strengths of 0.4~0.7 V/cm. These results indicate that the dcEFs can be focused on specific locations by suitably assigning the positive and negative electrodes (numbers and relative locations). This is helpful in conducting personalized GBM treatment.

**Figure 12:**
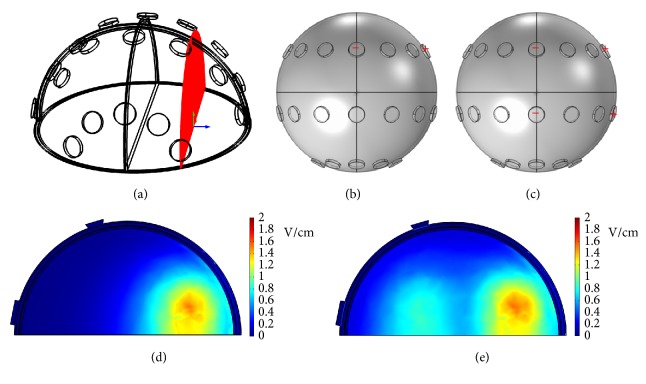
(a) The* y*-*z* plane cross-section located at* x* = 25 mm. (b) The electrode configuration where the rightmost electrode of the front array was assigned potentials and the center electrode of the front array was grounded. (c) The electrode configuration where the rightmost electrodes of the front and middle arrays were assigned potentials and the center electrodes of the front and middle arrays were grounded. (d) The dcEF distribution corresponding to the electrode configuration in (b). (e) The dcEF distribution corresponding to the electrode configuration in (c).

**Figure 13:**
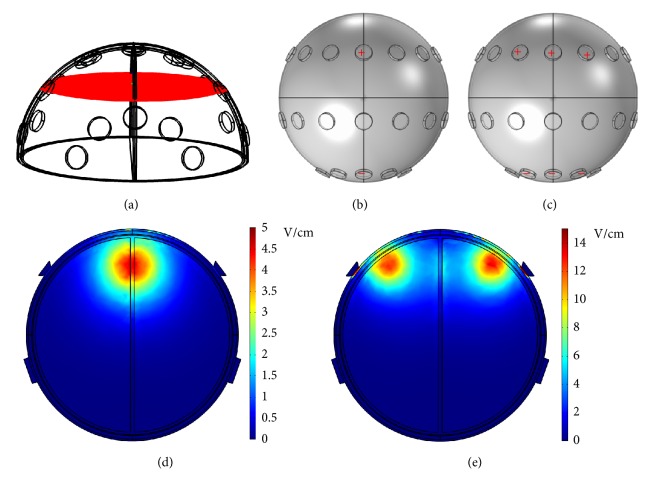
(a) The* x*-*y* plane cross-section located at* z* = 33 mm. (b) The electrode configuration where the center electrodes of the front and back arrays were assigned potentials and grounded, respectively. (c) The electrode configuration where the middle 3 electrodes of the front array were assigned potentials and the middle 3 electrodes of the back array were grounded. (d) The dcEF distribution corresponding to the electrode configuration in (b). (e) The dcEF distribution corresponding to the electrode configuration in (c).

To visualize the dcEF distributions from different points of view, various electrode configurations are tested.  Figure 12(a) shows the* y*-*z* plane cross-section located at* x* = 25 mm. In the electrode configuration where the rightmost electrode of the front array was assigned potentials and the center electrode of the front array was grounded (see Figure 12(b)), the dcEF distribution in that plane is displayed in Figure 12(d). At an applied potential of 100 V, the dcEFs are localized in the region between positive and negative electrodes. The dcEF strengths are around 0.6~1.4 V/cm. As the electrode configuration is changed to that in Figure 12(c) where the rightmost electrodes of the front and middle arrays were assigned potentials and the center electrodes of the front and middle arrays were grounded, the dcEF distribution is shown in Figure 12(e). Similarly, the dcEFs are distributed in regions between positive and negative electrodes, but the dcEF strengths in the middle array (0.6~0.8 V/cm, the center part of Figure 12(e)) are smaller than those in the front array (0.6~1.4 V/cm, the right part of Figure 12(e)).  Figure 13(a) shows the* x*-*y* plane cross-section located at* z* = 33 mm. In the electrode configuration where the center electrodes of the front and back arrays were assigned potentials and grounded, respectively (see Figure 13(b)), the dcEF distribution in that plane is displayed in Figure 13(d). At an applied potential of 100 V, the dcEFs are localized close to the positive electrode, with strengths of 2.5~4.5 V/cm.  Figure 13(c) shows another electrode configuration where the middle 3 electrodes of the front array were assigned potentials and the middle 3 electrodes of the back array were grounded.  Figure 13(e), displaying the dcEF distribution in that plane, indicates again that the dcEFs are localized close to the positive electrodes.

Finally, I investigate the total power dissipation density and temperature elevation due to Joule heating in different head tissues. The total power dissipation density (*P* in W/m^3^) is evaluated in each of the head tissues using the COMSOL Multiphysics software. The following equation is used:

(2) $$\begin{array}{c} Q=ms\Delta T. \end{array}$$

In this equation,* Q* is the electrically generated heat in the tissue,* m* is the mass of the tissue,* s* is the heat capacity of the tissue, and Δ*T* is the temperature elevation in the tissue. By using* m* =* DV*, where* D* is the density of the tissue, the following equation is derived:

(3) PVΔt=DVsΔT,  orΔTΔt=PDs.

The temperature increase per second can be calculated from total power dissipation density, density, and heat capacity.  Table 2 lists these values under an applied potential of 100 V in the electrode configuration shown in Figure 7(a). As indicated, the total power dissipation density in the scalp is 9.99 × 10^5^ W/m^3^, and this value decreases to 1.81 × 10^4^ W/m^3^ in the skull, to 2.42 × 10^3^ W/m^3^ in the dura, to 2.19 × 10^3^ W/m^3^ in the cerebrospinal fluid, and finally to 41.97 W/m^3^ in the brain. The corresponding temperature increases per second in these 5 tissues are 0.27, 6.54 × 10^−3^, 6.12 × 10^−4^, 5.31 × 10^−4^, and 1.11 × 10^-5°^C, respectively. For all tissues except the scalp, these increases are too small to be considered harmful. Since the scalp is in direct contact with the electrodes, a significant temperature elevation is noticed. This increase can be balanced via suitable conduction as the scalp is exposed to surrounding air at a constant room temperature.  Table 3 lists the total power dissipation densities and temperature increases per second of various brain tissues under an applied potential of 100 V in the electrode configuration shown in Figure 8(a). Temperature increases per second of 0.5, 0.012, 1.04 × 10^−3^, 6.6 × 10^−4^, and 1.23 × 10^-5°^C are calculated in the scalp, the skull, the dura, the cerebrospinal fluid, and the brain, respectively. To evaluate the maximum possible temperature increase, I employ a new electrode configuration where all electrodes were assigned potentials except the center one of the back array which was grounded.  Table 4 lists all simulated and calculated values. The temperature increases per second in these 5 tissues are 0.65, 0.016, 1.36 × 10^−3^, 8.53 × 10^−4^, and 1.62 × 10^-5°^C, respectively. This heat produced due to Joule heating can be easily dissipated via conduction, convection, and radiation from the scalp to the air. These results indicate that, under an applied voltage of 100 V, these EFs are thought to be harmless to the brain and all surrounding tissues.

## 4. Conclusion

In this paper, a 3D head model consisting of different head tissues was constructed to study the effects of applied potentials and electrode configurations on the dcEF distribution inside the brain. From the simulation results, the following findings are noticeable. First, an applied potential of 100 V is able to generate dcEF strengths of 0.5~1 V/cm inside the brain. These magnitudes are suitable for GBM treatment. Second, by suitably assigning the positive and negative electrodes (numbers and relative locations), the dcEFs can be focused on specific locations. This is helpful in conducting personalized electrotherapy. Finally, under an applied voltage of 100 V, a maximum possible temperature increase per second of 0.65°C is evaluated in the scalp. Therefore, these dcEFs are thought to be harmless to the brain and all surrounding tissues. These findings are believed to be useful in designing the electrode configuration for applications in GBM electrotherapy.

## Figures and Tables

**Table 1:** Dielectric properties, densities, and heat capacities of various brain tissues relevant to numerical simulations. Data obtained from the Foundation for Research on Information Technologies in Society (https://www.itis.ethz.ch/virtual-population/tissue-properties/database/dielectric-properties/).

	Conductivity^a^ *σ* (S/m)	Relative permittivity^a^ *ε*_*r*_	Density *D* (kg/m^3^)	Heat capacity *s* (J/kg°C)
Scalp	0.00105	1100	1109	3391
Skull	0.0529	295.5	1543	1793
Dura	0.502	290	1174	3364
Cerebrospinal fluid	2	109	1007	4096
Brain	0.108	1578	1043	3628

^a^at 200 kHz.

**Table 2:** Total power dissipation densities and temperature increases per second of various brain tissues under an applied potential of 100 V in the electrode configuration shown in Figure 7(a).

	Total power dissipation density (W/m^3^)	Temperature increase per second (°C/s)
Scalp	9.99 × 10^5^	0.27
Skull	1.81 × 10^4^	6.54 × 10^−3^
Dura	2.42 × 10^3^	6.12 × 10^−4^
Cerebrospinal fluid	2.19 × 10^3^	5.31 × 10^−4^
Brain	41.97	1.11 × 10^−5^

**Table 3:** Total power dissipation densities and temperature increases per second of various brain tissues under an applied potential of 100 V in the electrode configuration shown in Figure 8(a).

	Total power dissipation density (W/m^3^)	Temperature increase per second (°C/s)
Scalp	1.87 × 10^6^	0.5
Skull	3.39 × 10^4^	0.012
Dura	4.12 × 10^3^	1.04 × 10^−3^
Cerebrospinal fluid	2.74 × 10^3^	6.6 × 10^−4^
Brain	46.5	1.23 × 10^−5^

**Table 4:** Total power dissipation densities and temperature increases per second of various brain tissues under an applied potential of 100 V in the electrode configuration where all electrodes were assigned potentials except the center one of the back array which was grounded.

	Total power dissipation density (W/m^3^)	Temperature increase per second (°C/s)
Scalp	2.44 × 10^6^	0.65
Skull	4.43 × 10^4^	0.016
Dura	5.38 × 10^3^	1.36 × 10^−3^
Cerebrospinal fluid	3.52 × 10^3^	8.53 × 10^−4^
Brain	61.29	1.62 × 10^−5^
